# Adiponectin Decreases Gastric Smooth Muscle Cell Excitability in Mice

**DOI:** 10.3389/fphys.2019.01000

**Published:** 2019-08-06

**Authors:** Eglantina Idrizaj, Rachele Garella, Giovanni Castellini, Fabio Francini, Valdo Ricca, Maria Caterina Baccari, Roberta Squecco

**Affiliations:** ^1^Department of Experimental and Clinical Medicine, Section of Physiological Sciences, University of Florence, Florence, Italy; ^2^Psychiatric Unit, Department of Health Sciences, University of Florence, Florence, Italy

**Keywords:** adiponectin, gastric fundus, membrane properties, ion currents, satiety signals

## Abstract

Some adipokines known to regulate food intake at a central level can also affect gastrointestinal motor responses. These are recognized to be peripheral signals able to influence feeding behavior as well. In this view, it has been recently observed that adiponectin (ADPN), which seems to have a role in sending satiety signals at the central nervous system level, actually affects the mechanical responses in gastric strips from mice. However, at present, there are no data in the literature about the electrophysiological effects of ADPN on gastric smooth muscle. To this aim, we achieved experiments on smooth muscle cells (SMCs) of gastric fundus to find out a possible action on SMC excitability and on membrane phenomena leading to the mechanical response. Experiments were made inserting a microelectrode in a single cell of a muscle strip of the gastric fundus excised from adult female mice. We found that ADPN was able to hyperpolarize the resting membrane potential, to enhance the delayed rectifier K^+^ currents and to reduce the voltage-dependent Ca^2+^ currents. Our overall results suggest an inhibitory action of ADPN on gastric SMC excitation–contraction coupling. In conclusion, the depressant action of ADPN on the gastric SMC excitability, here reported for the first time, together with its well-known involvement in metabolism, might lead us to consider a possible contribution of ADPN also as a peripheral signal in the hunger–satiety cycle and thus in feeding behavior.

## Introduction

Adipokines are cytokines secreted by the white adipose tissue, able to influence a variety of physiological and pathophysiological processes through endocrine, paracrine, and autocrine mechanisms. ADPN, one of the most abundant adipokines secreted in the blood stream, regulates food intake by sending satiety signals at the central level, and exerts peripheral effects (Idrizaj et al., 2019). The observation that ADPN serum levels are correlated with body fat content and are lower in obese subjects (Bastard et al., 2006) has generated enormous interest within the scientific community (Henstridge and Febbraio, 2010). ADPN receptors, Adipo-R1 and Adipo-R2, have been originally identified in the hypothalamus (Scherer et al., 1995). Previous studies (Hoyda and Ferguson, 2010) showed that ADPN acts at a central level, controlling neuronal excitability of the hypothalamic paraventricular nucleus through the modulation of different K^+^ conductances and contributing to changes in membrane potential. Moreover, ADPN receptors have been found also in a variety of peripheral tissues (Yamauchi et al., 2003; Fasshauer et al., 2004; Kharroubi et al., 2004; Blüher et al., 2005; Liu et al., 2008; González et al., 2010; Hong et al., 2016) including the gastrointestinal tract (Idrizaj et al., 2018a). Acting through different signaling pathways, ADPN exerts antidiabetic, anti-inflammatory, antiatherogenic, and antiapoptotic effects (Idrizaj et al., 2019). Recently, other physiological roles of ADPN have emerged including the skeletal muscle sensitivity to this hormone (Krause et al., 2019), the prevention of cardiac dysfunctions (Francisco et al., 2016), and actions on the smooth muscle (AlSaif et al., 2015). Particularly, it has a proved vasorelaxant effect on vascular cells (Hong et al., 2016; Schinzari et al., 2017), and some of the mechanisms by which ADPN influences the contractile tone of small arteries have been clarified (Baylie et al., 2017). Besides vascular muscle activity, ADPN can influence that of the gastric one (Idrizaj et al., 2018a), but no effects of ADPN on the excitability of the SMCs of the gastrointestinal tract are reported at present. To this aim, we here intended to investigate the effect of this hormone on the bioelectric properties of SMC from the gastric fundus, focusing on the RMP, the ion currents responsible of the RMP control (Currò, 2014), and the voltage-dependent *I*_Ca_, mainly responsible for triggering the mechanical activity.

## Materials and Methods

The experimental procedure followed the European Community guidelines for animal care (DL 116/92, application of the European Communities Council Directive of 24 November 1986; 86/609/EEC) and was approved by the Committee for Animal Care and Experimental Use of the University of Florence in conformity with the *Guide for the Care and Use of Laboratory Animals* of the US National Institutes of Health (Idrizaj et al., 2018a, b). C57BL/6 (8–12 weeks old) female mice (Charles River, Lecco, Italy) were used (Squecco et al., 2013).

A muscular strip from the gastric fundus was pinned in a recording chamber (Squecco et al., 2013) bathed with a Krebs–Henseleit solution (mM): 118 NaCl, 4.7 KCl, 1.2 MgSO_4_, 1.2 KH_2_PO_4_, 25 NaHCO_3_, 2.5 CaCl_2_, and 10 glucose (pH 7.4). Intracellular recording was made by conventional microelectrode (resistance = 60–70 MΩ) inserted in a cell of the longitudinal smooth muscle layer and filled with the following internal solution (mM): 130 KCl, 10 NaH_2_PO_4_, 0.2 CaCl_2_, 1 ethylene-bis(oxyethylenenitrilo)tetraacetic acid (EGTA), 5 MgATP, and 10 4-(2-hydroxyethyl)-1-piperazineethanesulfonic acid (HEPES) (pH 7.2), unless otherwise stated. We used the Krebs–Henseleit solution as Ctrl external solution to record RMP and passive properties of SMCs. In order to record K^+^ current (*I*_K_) we used the Krebs–Henseleit as external solution with specific channel blockers such as Nifedipine (10 μM) for L-type *I*_Ca_, BaCl_2_ (0.4 mM) for eventual inward rectifier K^+^ current, 4-aminopyridine (4-AP, 2 mM) for eventual transient outward K^+^ current (Castle and Slawsky, 1993; Crescioli et al., 2008). According to Idrizaj et al. (2018a), to record only *I*_Ca_ we used a high-TEA external solution (mM): 10 CaCl_2_, 145 tetraethylammonium bromide, 10 HEPES, and a suitable filling pipette solution (mM): 150 CsBr, 5 MgCl_2_, 10 EGTA, and 10 HEPES (pH = 7.2). The current amplitude was normalized to cell capacitance, *C*_m_, to properly compare the currents recorded from cells of different size.

Recombinant full-length mouse ADPN was tested from 2 × 10^–11^ up to 10^–7^ M. Heptanol (1 mM) was consistently used to block gap junctional currents of the functional syncytium (Squecco et al., 2013). Drugs were from Sigma Chemical (St. Louis, MO, United States).

We recorded RMP of the SMCs before and after chemical stimulation in current clamp mode, with a stimulus waveform: *I* = 0 pA (Squecco et al., 2015). The membrane passive properties were consistently estimated in voltage clamp starting from a HP of −70 mV. *I*_K_ activation was elicited by 1-s long voltage pulses ranging from −80 to 50 mV applied in 10-mV increments (HP = −60 mV). *I*_Ca_ kinetics was analyzed as in Idrizaj et al. (2018b). Mathematical analysis of data was performed by pClamp6 (Axon Instruments). Statistical analysis was done using Student’s *t-*test or one-way ANOVA followed by Bonferroni’s *post hoc* test when more than two groups of data were compared. *n* represents the number of SMCs analyzed. Results are mean ± SEM. *P* ≤ 0.05 was considered significant unless otherwise specified.

## Results

### ADPN Hyperpolarizes the RMP of Gastric Fundus SMCs

**FIGURE 1:**
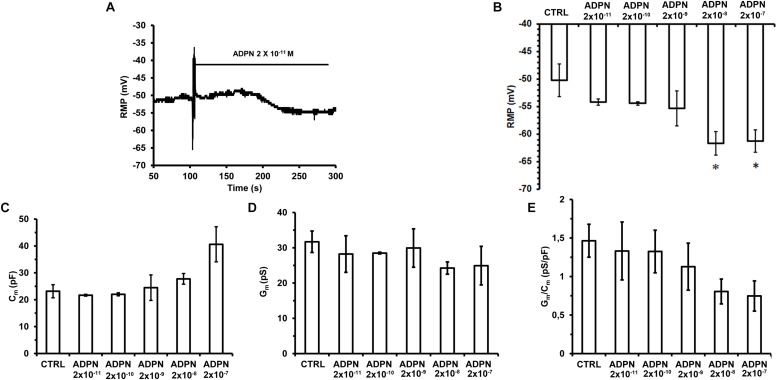
Effects of different ADPN concentrations on RMP and passive membrane properties of SMC from the gastric fundus. **(A)** Acute addition of ADPN to the bath solution (indicated by the horizontal line starting from the artifact) causes RMP hyperpolarization already appreciable with the lowest concentration used, namely 2 × 10^– 11^ M. **(B)** The statistically significant hyperpolarization was induced by ADPN 2 × 10^– 8^ M (*P* = 0.0068). **(C–E)** Data evaluated in Ctrl condition and 5 min after the addition of different ADPN concentrations to the bath solution. **(C)** Cell capacitance (*C*_m_) as an index of cell surface. **(D)** Membrane conductance as an index of resting permeability (*G*_m_). **(E)** *G*_m_/*C*_m_. Values are means ± SEM. One-way ANOVA with repeated measures was used for multiple comparisons followed by Bonferroni’s *post hoc* test. ^*^P < 0.05 vs. Ctrl. Ctrl *n* = 50, ADPN *n* = 35 (10 mice).

We first evaluated the effects of ADPN on the RMP of SMCs to assess its possible impact on cell excitability. Acute ADPN addition to the bath solution caused a hyperpolarization already appreciable at the lowest doses employed (2 × 10^–11^ M) that reached the maximal value in about 3 min (Figure 1A). However, only starting from 2 × 10^–8^ M we observed a hyperpolarization statistically different compared to the RMP of the Ctrl cells. Higher doses did not cause further hyperpolarization (Figure 1B). This hyperpolarizing effect may indeed concur to hinder the SMC excitability (Squecco et al., 2015; Idrizaj et al., 2018b).

### Effects of ADPN on the Membrane Passive Properties of Gastric Fundus SMCs

To estimate possible modifications of the SMC membrane passive properties, we first measured the *C*_m_ value in Ctrl condition and 10 min after the addition of ADPN to the external bath solution. Compared to the Ctrl values, ADPN induced a slight augmentation of *C*_m_ starting from a concentration of 2 × 10^–9^ M, that became progressively higher as the dose increased, although not statistically significant for any concentration used (Figure 1C). The analysis of the *G*_m_ (Figure 1D) and of the specific conductance, *G*_m_/*C*_m_, in the presence of ADPN (Figure 1E) revealed a tendency to become smaller compared to the Ctrl values starting from 2 × 10^–8^ M, indicating that ADPN scarcely affected the SMC membrane properties.

### ADPN Increases *I*_K_ and Decreases *I*_Ca_ Amplitude in Gastric Fundus SMCs

**FIGURE 2:**
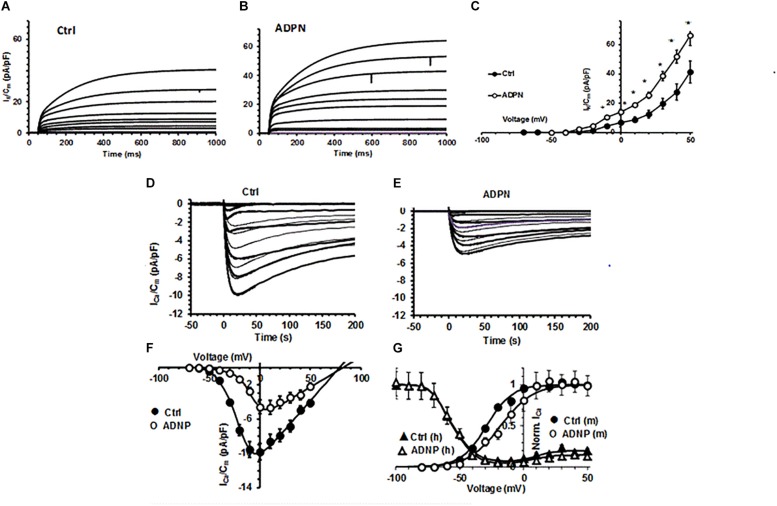
Effects of ADPN on voltage-dependent K^+^ current and *I*_Ca_ recorded in a SMC from the gastric fundus. **(A,B)** Representative total outward K^+^ currents (*I*_K_) recorded in Ctrl **(A)** and in the presence of ADPN (2 × 10^– 8^ M) **(B)**, elicited by voltage steps from –80 to 50 mV (HP = –60 mV). **(C)** *I–V* plots related to *I*_K_ in Ctrl (filled circles) and in the presence of ADPN (open circles). **(D)** Typical *I*_Ca_ traces obtained in a Ctrl **(D)** and ADPN (2 × 10^– 8^ M) treated **(E)** SMCs in response to 4-s voltage pulses from –70 to 50 mV (HP = –80 mV), in 10-mV increments, in high-TEA bath solution. **(F)** *I*–*V* plots related to *I*_Ca_ in Ctrl (filled circles) and ADPN-treated SMCs (open circles). All the ADPN data points are statistically different (*P* < 0.05) to Ctrl over *I*_Ca_ threshold. **(G)** Steady state activation (m) and inactivation (h) analysis of normalized *I*_Ca_: effect of ADPN (open circles) compared to Ctrl (filled circles) and lack of effects on inactivation (triangles). The continuous lines through the experimental data represent the fitted Boltzmann function. Note that, at positive potentials, the inactivation curve is *U*-shaped and decreased by ADPN. Current values are normalized to *C*_m_. All of the data are mean values ± SEM. Differences with *P* < 0.05 were considered significant: ^*^P < 0.05, ^∗∗^*P* < 0.01, ^∗∗∗^*P* < 0.001 (Student’s *t*-test). Statistical significance is not depicted in the figure for clarity but is reported for the various Boltzmann parameters in Supplementary Table 1. Data are from 18 to 20 cells (four mice).

Trying to explain the observed membrane hyperpolarization, we tested the effects of ADPN on the main voltage-dependent *I*_K_ commonly supposed to Ctrl the RMP. Since the major effect of ADPN on the RMP was obtained at the concentration of 2 × 10^–8^ M, we used this dose for all the following experiments. As expected, ADPN treatment increased *I*_K_ compared to Ctrl (Figures 2A–C) and this can undoubtedly contribute to membrane hyperpolarization.

Aiming to investigate if ADPN could affect the first steps of electro-mechanical coupling, we also evaluated its effect on *I*_Ca_. In Ctrl preparations, we constantly recorded inward current from the SMC resembling smooth muscular *I*_Ca_ (Figure 2D) with the 0-mV step pulse evoking the maximal *I*_Ca_ amplitude. The acute addition of ADPN (2 × 10^–8^ M) to the external bath solution decreased this current amplitude and caused a different voltage dependence. In fact, the maximal peak size was reached with the 10-mV step pulse in the presence of ADPN (Figure 2E). The *I*–*V* curve analysis confirmed this general behavior (Figure 2F). We also performed the steady state analysis of the *I*_Ca_ activation and inactivation curves that were best-fitted by the Boltzmann function (Figure 2G): ADPN added to the bath solution strongly reduced the current size but did not affect the voltage dependence of its inactivation, whereas that of activation was positively shifted. The related Boltzmann parameters with the statistical significance are listed in Supplementary Table 1. These earliest results on *I*_Ca_ indicate that ADPN modulates Ca^2+^ influx altering the voltage-dependent channel kinetics in the gastric SMC.

## Discussion

Some adipokines that act at the central level to influence feeding behavior seem to affect gastrointestinal motor phenomena, which represent peripheral signals involved in the regulation of food intake (Duca and Covasa, 2012). In this view, leptin (Yarandi et al., 2011) and more recently ADPN appear to influence gastrointestinal motility in addition to their central actions. Particularly, ADPN is able to induce a decrease of the gastric mechanical activity in mice (Idrizaj et al., 2018a). The present results indicate for the first time that the hormone can influence the gastric SMCs’ electrophysiological properties, which represent the first steps for the mechanical responses. Indeed, in keeping with our previous observation that ADPN induced gastric relaxation (Idrizaj et al., 2018a), we note that the hormone strongly influences SMCs’ excitability by inducing membrane hyperpolarization. This effect can be determined, at least in part, by the here-observed tendency toward the reduction of *G*_m_, since this may hamper the aspecific entry of depolarizing ions, leading to a decreased SMC excitability. Moreover, we also noted that ADPN induces an increase of *I*_K_, which is known to play an important role in RMP Ctrl. Although this effect was not extraordinarily broad, it may indeed contribute to the hyperpolarization. A more negative RMP definitely disturbs the related electromechanical coupling since a more intense stimulus than usual is required to activate high voltage threshold-operated ionic channels (Dwyer et al., 2011; Idrizaj et al., 2018a). Accordingly, it became remarkable to study ADPN effect also on *I*_Ca_. In fact, this current represents a chief source for intracellular Ca^2+^ elevation useful for contractile activation and its eventual modifications may further affect the SMC mechanical activity. Interestingly, we found that ADPN reduced *I*_Ca_ amplitude exerting an inhibitory effect on Ca^2+^ influx through voltage-dependent Ca^2+^ channels, further supporting its influence in hindering the SMC electromechanical coupling.

However, our study raises several additional queries needing further investigation, such as the type of K^+^ channel mostly involved ADPN effects, and the possible signaling pathways through which ADPN modulates the gastric SMC excitability. To answer these questions, further studies are in progress in our laboratory. Several signaling paths have been reported in relation to non-gastric smooth muscle and other targets for ADPN such as AMP-activated protein kinase (AMPK), peroxisome proliferative-activated receptor (PPAR)-α expression, ceramidase activity, and sphingosine 1 phosphate (S1P) formation (Botta et al., 2019; Kim and Park, 2019). This and some other previous reports dealing with NO signaling (Chen et al., 2003; Grossini et al., 2016; Nour-Eldine et al., 2016) will provide a useful background for our future studies.

## Conclusion

In conclusion, this preliminary study offers the first evidence that ADPN exerts a novel inhibitory function at the SMC plasma membrane level in gastric preparations that concurs to an actual weakened SMC excitability (Koh et al., 2012). ADPN seems to hinder the first steps of the excitation–contraction coupling, which is in perfect agreement with our previously published mechanical findings (Idrizaj et al., 2018a). Thus, ADPN seems to favor gastric muscle relaxation, which may lead to a consequent increase of organ capacity. Because gastric distension represents, from a physiological point of view, a peripheral satiety signal, we speculated that the here-observed peripheral effects are part of a control system designed to regulate food intake, which might concur to suppress feeding behavior. These observations provide a stimulating background to the challenging hypothesis that ADPN and/or its receptors could be a potential therapeutic tool in the treatment of obesity (Li et al., 2017) and eating disorders and, certainly, this issue deserves further investigation in a translational perspective.

## Ethics Statement

This study was carried out in accordance with the recommendations of the European Community guidelines for animal care (DL 116/92, application of the European Communities Council Directive of 24 November 1986; 86/609/EEC). The protocol was approved by the Committee for Animal Care and Experimental Use of the University of Florence in conformity with the Guide for the Care and Use of Laboratory Animals of the US National Institutes of Health.

## Author Contributions

EI and RS performed the electrophysiological experiments. EI, RG, FF, and RS analyzed the data. EI, RS, and FF prepared the figures. RS, MB, GC, and VR designed the research study. RS wrote the manuscript. RS, EI, MB, FF, RG, GC, and VR critically revised the manuscript.

## Conflict of Interest Statement

The authors declare that the research was conducted in the absence of any commercial or financial relationships that could be construed as a potential conflict of interest.

## Abbreviations

ADPN: adiponectin
*C*_m_: cell linear capacitance
Ctrl: control
*G*_m_: membrane conductance
*G*_m_/*C*_m_: specific membrane conductance
HP: holding potential
*I*_*a*_: current activation
*I*_Ca_: Ca^2+^ current
*I*_K_: voltage-dependent delayed rectifier K^+^ currents
*k*_*a*_: steepness factor of activation
*k*_h_: steepness factor of inactivation
RMP: resting membrane potential
SMC: smooth muscle cell
*V*_*a*_: half-maximal activation voltage
*V*_h_: half-maximal inactivation voltage
*V*_rev_: apparent reversal potential.
