# Supplementary material for: Adiponectin Decreases Gastric Smooth Muscle Cell Excitability in Mice
Source: Front Physiol. 2019 Aug 6;10:1000. doi: 10.3389/fphys.2019.01000 (PMC6691180; doi:10.3389/fphys.2019.01000)
Supplement: Supplementary file 1 [file Table_1.docx]

**Table 1. Boltzmann parameters of *I*_Ca_ activation and inactivation in control (Ctrl) and in ADPN-treated (ADPN) smooth muscle cells from gastric fundus.**

| **Parameters** | **Ctrl** | **ADPN**  **(2 x 10^-8^ M)** |
| --- | --- | --- |
| *I*_Ca,p_ /*C*_m_ (pA/pF) | 10.2 ± 1.4 | 5.1 ± 0.5*** |
| *G*_m_/*C*_m_ (pS/pF) | 52.4 ± 5.1 | 32.4 ± 3.8*** |
| *V*_thr_ (mV) | -58.2 ± 3.1 | -50.7 ± 3.3** |
| *V*_p_ (mV) | 0.1 ± 0.07 | 5.2 ± 1.3*** |
| *V*_a_ (mV) | -29.5 ± 1.7 | -18.7 ± 1.3*** |
| *k*_a_ (mV) | 9.6 ± 0.6 | 12.5 ± 0.8* |
| *V*_rev_ (mV) | 78.6 ± 6.4 | 81.4 ± 7.5 |
| *V*_h_ (mV) | -50.9 ± 4.5 | -52.7 ± 5.4 |
| *k*_h_ (mV) | 7.5 ± 0.5 | 7.5 ± 0.5 |
| *t*_p_ (ms) | 22.7 ± 2 | 21.8 ± 3 |

The following equations:

$Ia(V) = Gmax (V-Vrev)/\{1 + exp[(Va-V)/ka]\}$ and $Ih(V) = I/\{1 + exp[-(Vh-V)/kh]\}$ were used to study the steady-state activation and inactivation curves. G_max_ represents the maximal conductance for *I*_a_; *V*_rev_ is the apparent reversal potential; *V*_a_ and *V*_h_ are the voltages causing the half-maximal activation and inactivation, respectively; *k*_a_ and *k*_h_ are the steepness factors of activation and inactivation, respectively.

Boltzmann parameters for *I*_Ca_ evaluated by fitting the single Boltzmann function through the experimental data are listed here. The subscript ‘p’ indicates the peak value. *I*_Ca,p_/*C*_m_ is the peak current amplitude normalized for cell capacitance; *G*_m_/*C*_m_ is the specific maximal conductance; *V*_thr_ is the voltage threshold; *V*_p_ is the voltage eliciting the maximal or peak current; *V*_a_ and *V*_h_ are the voltages causing the half-maximal current activation and inactivation, respectively; *k*_a_ and *k*_h_ are the steepness factors of activation and inactivation, respectively; *V*_rev_ is the apparent reversal potential; *t*_p_ is the time to peak of the *I*_Ca_ elicited by the 0- mV pulse. Differences with *P* < 0.05 were considered significant (Student’s t test): * for *P* < 0.05, ** for *P*  < 0.01, *** for *P*  < 0.001. Data are from 18-20 cells (4 mice).
